# Regulation of Retinoschisin Secretion in Weri-Rb1 Cells by the F-Actin and Microtubule Cytoskeleton

**DOI:** 10.1371/journal.pone.0020707

**Published:** 2011-06-27

**Authors:** Eiko Kitamura, Yekaterina E. Gribanova, Debora B. Farber

**Affiliations:** 1 Jules Stein Eye Institute and Department of Ophthalmology, David Geffen School of Medicine, University of California Los Angeles, Los Angeles, California, United States of America; 2 Molecular Biology Institute, University of California Los Angeles, Los Angeles, California, United States of America; 3 Brain Research Institute, University of California Los Angeles, Los Angeles, California, United States of America; University Medical Center Groningen, University of Groningen, Netherlands

## Abstract

Retinoschisin is encoded by the gene responsible for X-linked retinoschisis (XLRS), an early onset macular degeneration that results in a splitting of the inner layers of the retina and severe loss in vision. Retinoschisin is predominantly expressed and secreted from photoreceptor cells as a homo-oligomer protein; it then associates with the surface of retinal cells and maintains the retina cellular architecture. Many missense mutations in the *XLRS1* gene are known to cause intracellular retention of retinoschisin, indicating that the secretion process of the protein is a critical step for its normal function in the retina. However, the molecular mechanisms underlying retinoschisin's secretion remain to be fully elucidated. In this study, we investigated the role of the F-actin cytoskeleton in the secretion of retinoschisin by treating Weri-Rb1 cells, which are known to secrete retinoschisin, with cytochalasin D, jasplakinolide, Y-27632, and dibutyryl cGMP. Our results show that cytochalasin D and jasplakinolide inhibit retinoschisin secretion, whereas Y-27632 and dibutyryl cGMP enhance secretion causing F-actin alterations. We also demonstrate that high concentrations of taxol, which hyperpolymerizes microtubules, inhibit retinoschisin secretion. Our data suggest that retinoschisin secretion is regulated by the F-actin cytoskeleton, that cGMP or inhibition of ROCK alters F-actin structure enhancing the secretion, and that the microtubule cytoskeleton is also involved in this process.

## Introduction

Retinoschisin, also known as RS1, is a retinal-specific protein encoded by the *XLRS1* gene. Mutations in this gene cause X-linked retinoschisis (XLRS), a leading cause of macular degeneration in juvenile male patients [1]. The XLRS disease is characterized by areas of schisis in the macula at the level of the nerve fiber and ganglion cell layers – splitting that results in the formation of cystic cavities in the central retina – and by a reduced b-wave amplitude in the electroretinogram (ERG). These defects lead to impaired visual signal processing and progressive vision loss with age [2].

Following the identification of the murine ortholog of *XLRS1*, *Xlrs1* or *Rs1h* [3], [4], knockout mice deficient in this gene were generated [5]. These mice showed disruption of the cell layer architecture of the retina, irregular displacement of photoreceptor cells to other retinal layers and increased extracellular space in the region of photoreceptor ribbon synapses [5]. Subretinal delivery of the *Rs1h* or *XRLS1* gene into the knockout mice restored retinal structure and function, indicating that retinoschisin plays an important role in maintaining the proper architecture of the retina and the integrity of the optic nerve fiber layer [6]–[8]. Other studies also showed that retinoschisin serves as an anchor protein for the maintenance of the organization of retinal synapses, especially the photoreceptor synapses [9], [10]. After assembly into disulfide-linked multimers, retinoschisin interacts with proteins and phospholipids at the surface of photoreceptors and other cell types forming multi-molecular complexes with extracellular and cytoplasmic proteins. These complexes may constitute the stabilizing scaffold for the synapses [11]–[14].

Retinoschisin consists of a 23-amino acid N-terminal signal peptide, a 41-amino acid retinoschisin- specific domain, which is well conserved across species, and a 157-amino acid discoidin domain flanked by two small segments of 39 and 5 amino acids [11]. The signal peptide plays an essential role in guiding the nascent retinoschisin polypeptide to the lumen of the ER and it is subsequently removed by a signal peptidase. The folded peptide assembles into a disulfide-linked homo-octameric complex prior to secretion from cells [11]. It has been shown that Weri-Rb1 retinoblastoma cells express and secrete retinoschisin [15] and that in adult mouse retina, retinoschisin is primarily secreted from the inner segments of photoreceptors and, to a lesser extent, from bipolar cells [9], [10], [13], [15]. Wang et al. [16] described that missense mutations in the signal peptide or discoidin domain lead to intracellular retention of mutant retinoschisins, implicating the corresponding mutated amino acids in the molecular mechanisms underlying retinoschisin secretion. Moreover, it has been reported recently that retinoschisin secretion is under circadian control in chick retina [17]. However, little is known about the intracellular regulatory factors that regulate retinoschisin transit and ultimately its exit from the photoreceptor cells.

The cytoskeleton, which consists of actin filaments and microtubules, is a highly dynamic network actively involved in many essential biological mechanisms such as those regulating cellular structure, transport, cell movement, differentiation, and signaling. Numerous studies have reported the requirement for an intact cytoskeleton for the process of secretion [18], [19]. In this respect, Rho, one of the members of the small G-protein family, and its effector, the Rho-associated kinase (ROCK) have been shown to participate in the actin cytoskeleton assembly and reorganization, and in a variety of other cellular processes including cell contractility, cell-cell adhesion, migration and invasion, phagocytosis, apoptosis and exocytosis [20]. Here we show that depolymerization as well as hyperpolymerization of F-actin inhibits retinoschisin secretion, resulting in the accumulation of retinoschisin within cells. On the other hand, relaxing or loosening the F-actin cytoskeleton by inhibition of ROCK enhances retinoschisin secretion. Thus, our data define a dual role for the F-actin cytoskeleton as a regulator of retinoschisin secretion (inhibitor or enhancer) and demonstrate a critical involvement of ROCK in this process. We also show that disorganization of microtubules reduces retinoschisin secretion, suggesting an active participation of this other component of the cytoskeleton in the release of retinoschisin from retinal cells.

## Results

### Retinoschisin co-localizes with F-actin and the microtubule cytoskeleton

**Figure 1:**
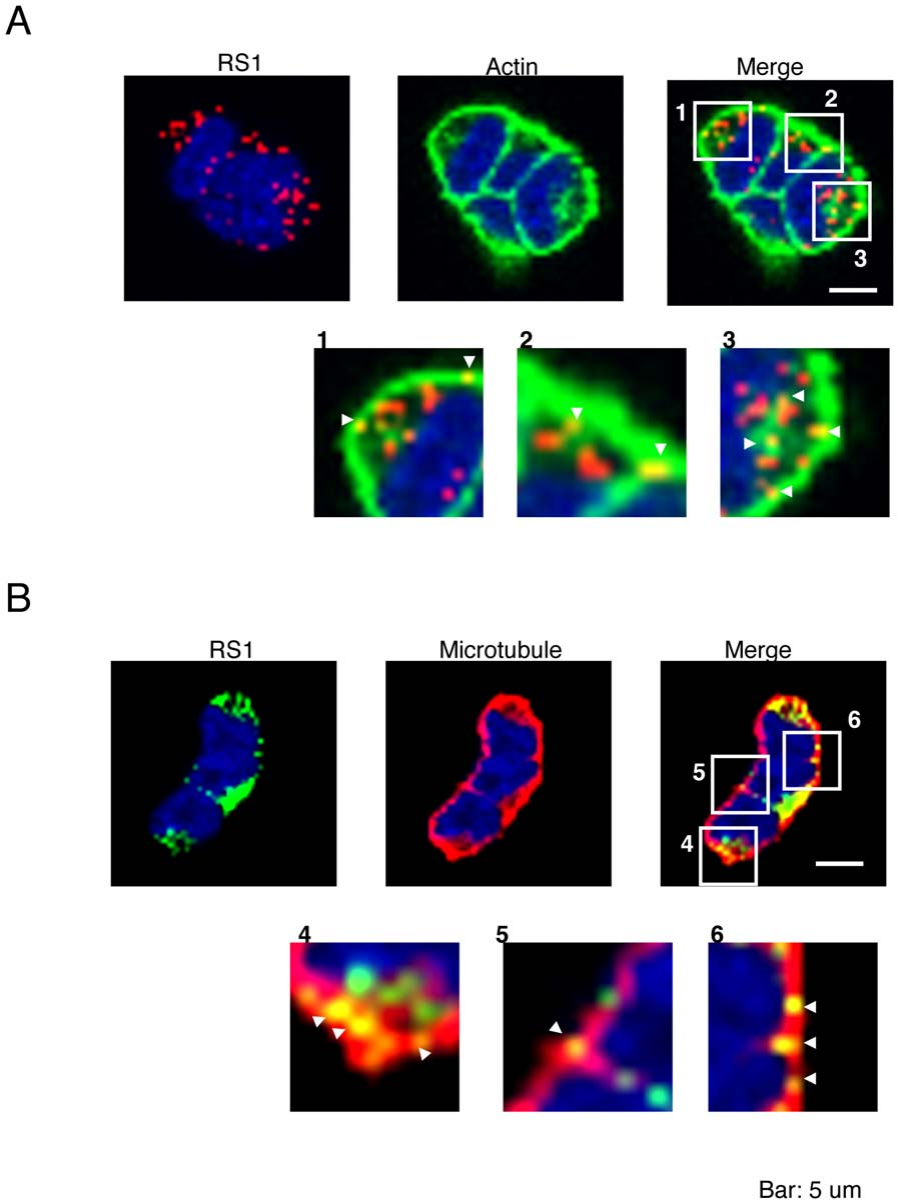
Confocal microscopy images of Weri-Rb1 cells. (A) Cells were double-stained using the rabbit RS24-37 retinoschisin antibody followed by Cy3-conjugated donkey-anti-rabbit IgG (RS1, red) and Alexa Fluor 488-phalloidin for F-actin (green). DAPI labeled the nuclei (blue). The right image corresponds to the merged RS1 and F-actin images. The white squares limit Areas 1, 2 and 3 that have been zoomed in below. (B) Cells were double-stained for retinoschisin with RS24-37 antibody followed by Alexa Fluor 488 goat anti-rabbit IgG (green) and alpha tubulin antibody followed by Alexa Fluor 568 goat anti-mouse IgG (red). Nuclei were labeled with DAPI (blue). A merged image of the cells labeled for RS1 and alpha-tubulin is at the right. Retinoschisin co-localized with the cytoskeleton proteins F-actin and alpha-tubulin as indicated by the arrowheads in the enlarged images (1–6). Bar, 5 µm.

In order to define whether F-actin is involved in retinoschisin secretion, Triton X-100 permeabilized cells were stained with the high-affinity probe for F-actin, Alexa Fluor 488-phalloidin, and RS24-37. Weri-Rb1 cells are small, round cells with thin cytoplasm and large nuclei; they grow in grape-like clusters [21]. Retinoschisin vesicles localized to their cytoplasm and cell membrane, but not to their nuclei (Fig. 1, A and B). F-actin was seen surrounding the nuclei and along the cells' membranes as well as in the cytoplasm (Fig. 1A). Partial co-localization of retinoschisin with F-actin was observed in small, dot-like yellow structures (Fig. 1A, enlarged areas 1, 2 and 3). We also double-stained cells using antibodies against alpha tubulin and retinoschisin. Microtubules were detected uniformly throughout the cells as dense staining (Fig. 1B) and they partially co-localized with retinoschisin (Fig. 1B, enlarged areas 4, 5 and 6). The co-localization of retinoschisin with F-actin or microtubules suggests that the cytoskeleton has the potential to regulate retinoschisin secretion.

### Cytochalasin D inhibits retinoschisin secretion

**Figure 2:**
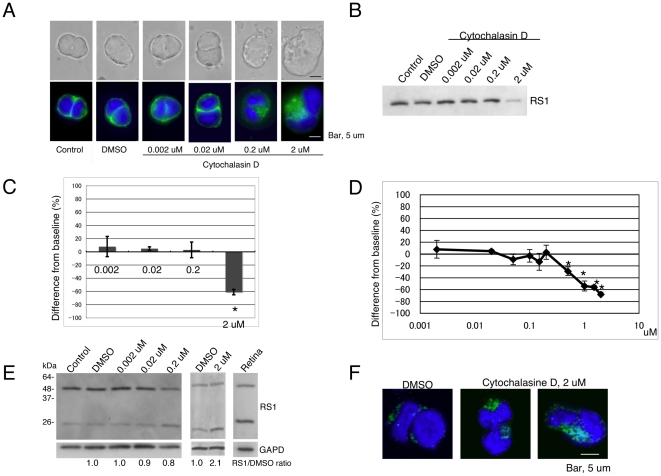
Retinoschisin secretion into the conditioned medium is decreased by addition of cytochalasin D. (A) Effects of cytochalasin D on cell morphology and F-actin cytoskeleton organization are dose dependent. Weri-Rb1 cells were cultured for 72 h in medium alone (untreated control) and medium containing DMSO or the indicated concentrations of cytochalasin D. Cells were then fixed, labeled with Alexa Fluor 488-phalloidin (green) and DAPI, and examined for changes in F-actin cytoskeleton organization. The upper row of pictures shows transmitted light images and the lower row, fluorescent light images. Bar, 5 µm. (B) Retinoschisin secretion into the conditioned medium decreases by treatment of cells with 2 µM cytochalasin D. Cells were treated as in (A) for 72 h. The medium for each condition was analyzed by Western blot using the RS24-37 antibody against retinoschisin. (C) Quantification of the intensity of each band on the Western blot in (B) (see MATERIALS AND METHODS). The data are means ± standard error of the mean (SEM) of three biological samples analyzed for each condition. (*) indicates that a p<0.05 was obtained for the difference between 2 µM cytochalasin D- and DMSO-treated cells. (D) Retinoschisin secretion decreases in a dose-dependent manner by treatment of cells with cytochalasin D at concentrations between 0.5 µM and 2 µM. Retinoschisin secretion was examined in the media of cells treated with cytochalasin D concentrations in the range of 0.02 to 2 µM as in (B) and quantified as stated in (C). (E) Retinoschisin level in the whole cell lysate increases with 2 µM cytochalasin D treatment. After 72 hr incubation in medium alone, medium containing DMSO or the indicated concentrations of cytochalasin D (left and middle panels) the cells were collected and analyzed by Western blot. Both the monomer and dimer of retinoschisin were detected in the retinal lysate (right panel). The intensities of the dimer and monomer bands for each sample were added and normalized to GAPDH. The normalized results were compared to the values obtained for the cells incubated in medium containing only DMSO and are shown as RS1/DMSO ratios. (F) Retinoschisin accumulates within cells treated with 2 µM cytochalasin D. Cells were treated with DMSO or 2 µM cytochalasin D for 72 h. Cells were then fixed and incubated with RS24-37 antibody, followed by Alexa Fluor 488 goat anti-rabbit IgG. Nuclei were labeled with DAPI.

To investigate the role of F-actin in retinoschisin secretion, we treated Weri-Rb1 cells with cytochalasin D, which depolymerizes the F-actin cytoskeleton network. The dose-dependent effect of this drug on cellular F-actin was visualized using Alexa Fluor 488 phalloidin (Fig. 2A). Concentrations up to 0.02 µM cytochalasin D did not produce distinct morphological changes in the cells, which displayed a smooth F-actin cytoskeleton such as that observed in control, non-treated cells or in cells treated only with DMSO. At a concentration of 0.2 µM cytochalasin D, cells were swollen and F-actin was observed as small puncta, indicating fragmentation of the F-actin cytoskeleton, but cells were still surrounded by thin F-actin filaments. A higher concentration of cytochalasin D (2 µM) caused more swelling and led to the aggregation of F-actin in dense patches; in addition, the thin filaments surrounding the cells were no longer seen. Overall, the changes in the F-actin cytoskeleton of Weri Rb1 cells produced by cytochalasin D are similar to those previously reported to occur in HEp-2 cells subjected to cytochalasin D treatment [22]. To evaluate the effect of the drug on retinoschisin secretion, Weri Rb1 cells were incubated with cytochalasin D at the concentrations used in Figure 2A, and the retinoschisin secreted into the respective conditioned medium was estimated after Western blotting (Figs. 2B and 2C). Our results indicated that DMSO, as well as 0.002 and 0.02 µM cytochalasin D, had no effect on cell morphology, the actin cytoskeleton organization, and retinoschisin secretion (Figs. 2 A, B and C). Even when the F-actin cytoskeleton was fragmented at 0.2 µM cytochalasin D, retinoschisin secretion was similar to that observed with only DMSO present in the medium. On the other hand, retinoschisin secretion was significantly decreased at 2 µM cytochalasin D, a concentration of the drug that caused severe disruption of the F-actin cytoskeleton. This finding indicated that F-actin, but not an intact F-actin cytoskeleton, is required for retinoschisin secretion.

High concentrations of actin-disrupting drugs have previously been shown to inhibit secretory or exocytotic events, while low concentrations facilitate these events [23]–[25]. To determine whether there was a biphasic response for retinoschisin secretion after cytochalasin D treatment of cells and if so, which were the critical concentrations of the drug that elicited these responses, we examined the effects of nine different concentrations of cytochalasin D between 0.02 to 2 µM (Fig. 2D); 5 other concentrations between 0.002 and 0.02 µM did not change the secretion level (data not shown). No biphasic response was observed within this range of concentrations. However, our results indicated that retinoschisin secretion decreased in a dose-dependent manner between 0.5 and 2 µM cytochalasin D (Fig. 2D).

We next examined whether retinoschisin accumulated in the cells exposed to cytochalasin D at concentrations of 0.002, 0.02 and 0.2 µM (Fig. 2E, left panel) and at 2 µM (Fig. 2E, middle panel). Western blots of the proteins from the different cell lysates using the RS24-37 antibody showed both the retinoschisin monomer and dimer even after reduction by DTT, as was previously observed with retinal samples [15], [10], (Fig. 2E, right panel). Pre-absorbing the RS24-37 antibody with the peptide that was used to generate it corroborated its specificity: neither the monomer nor the dimer bands were detected. As shown in Figures 2E (2.1±0.4 –fold) and 2F, accumulation of retinoschisin was observed within cells treated with 2 µM cytochalasin D.

**Figure 3:**
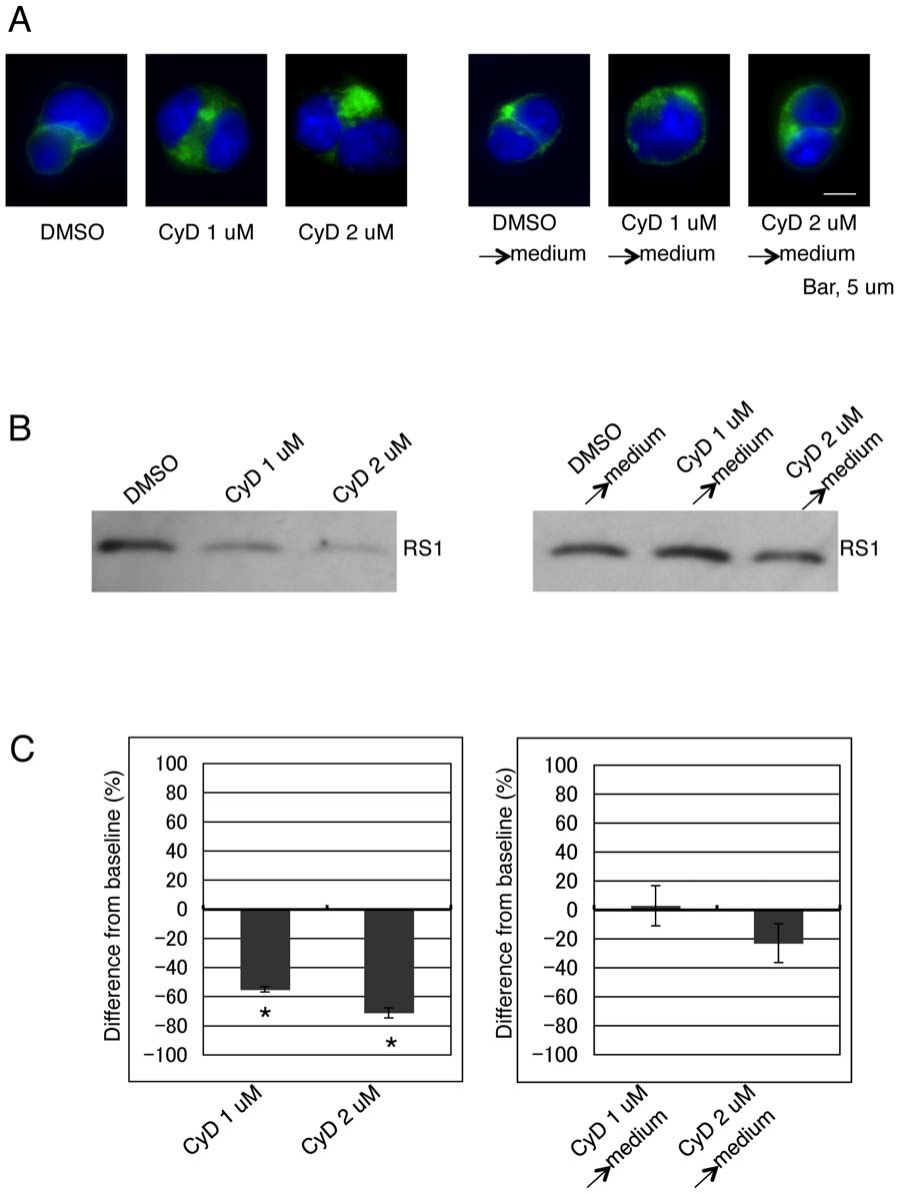
The effect of cytochalasin D on retinoschisin secretion is reversible. (A) F-actin cytoskeleton organization visualized with Alexa Fluor 488-phalloidin (green). Duplicate cell samples were treated with DMSO or 1 µM and 2 µM cytochalasin D (CyD) for 72 hr. One of the sets of cells was then fixed and labeled for F-actin (left panel). The conditioned medium of the duplicate set of samples was replaced by fresh medium without DMSO (DMSO→medium) or cytochalasin D (CyD 1 or 2 µM→medium) in it. Cells were incubated for another 72 hr in the fresh medium, fixed and labeled with Alexa Fluor 488-phalloidin. (B) Western blot of proteins from the medium of each sample described in (A). Retinoschisin was detected using the RS24-37 antibody. (C) Quantification of the intensity of each band on the Western blot in (B) (see MATERIALS AND METHODS). The data are means ± SEM of three different biological samples for each condition. *: p<0.05 was obtained for the difference between 1 µM or 2 µM cytochalasin D- and DMSO-treated cells.

We also investigated the reversibility of the cytochalasin D effect. After exposure to DMSO and 1 µM or 2 µM cytochalasin D for 72 hr, cells were cultured for another 72 hr in fresh medium that had no drug in it. Removal of 1 µM cytochalasin D from the cell culture restored the F-actin structure from dense patches to small puncta and allowed thin filaments to surround again the cells (Fig. 3A, right panel), a morphology very similar to that observed in cells treated with 0.2 µM cytochalasin D (Fig. 2A). Furthermore, retinoschisin secretion was fully restored to the level produced by DMSO (Figs. 3B and 3C, right panels). Removal of 2 µM cytochalasin D from the medium restored the thin filaments surrounding the cells, but dense patches still remained in the cytoplasm (Fig. 3A, right panel). Although secretion was restored, it was 20% lower than that of cells incubated only with DMSO (Figs. 3B and 3C, right panels). These results indicate that the effect of cytochalasin D on retinoschisin secretion is reversible and that the degree of recovery is dose-dependent.

### Jasplakinolide inhibits retinoschisin secretion

**Figure 4:**
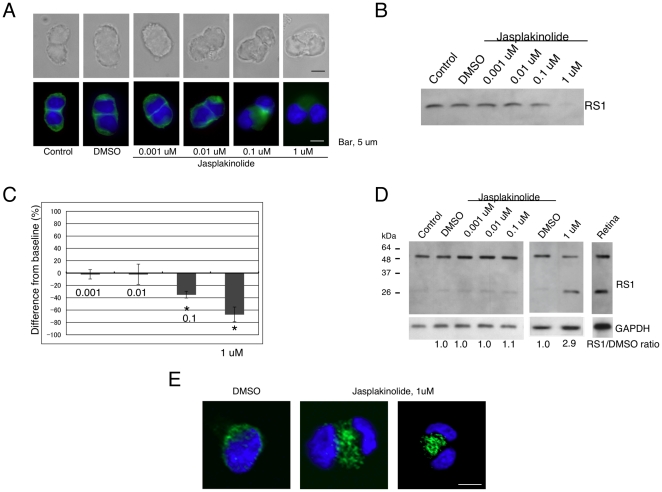
Retinoschisin secretion into the conditioned medium is decreased by addition of jasplakinolide. (A) Effects of jasplakinolide on cell morphology and F-actin cytoskeleton organization. Weri-Rb1 cells were cultured in medium alone (untreated control) and medium containing DMSO or the indicated concentrations of jasplakinolide for 72 h. Cells were then fixed and labeled with Alexa Fluor 488-phalloidin (green). The upper row of pictures shows transmitted light images and the lower row, fluorescent light images. Bar, 5 µm. (B) Western blot of proteins in the conditioned media of cells treated with DMSO or the indicated concentrations of jasplakinolide for 72 h using the RS24-37 antibody. As seen, retinoschisin secretion into the conditioned medium is decreased by 1 uM jasplakinolide. (C) Quantification of the intensity of each band in the Western blot in (B) (see MATERIALS AND METHODS). The data are means ± SEM of three different biological samples for each condition. *: p<0.05 was obtained for the difference between 1 µM jasplakinolide- and DMSO-treated cells. (D) Restinoschisin accumulates inside cells treated with 1 µM jasplakinolide. After 72 hr incubation in medium alone, medium containing DMSO or the indicated concentrations of jasplakinolide, cells were collected and analyzed by Western blot. The intensities of the dimer and monomer bands for each condition were added and normalized to GAPDH. The normalized values were then compared to that of DMSO-treated cells and are shown as RS1/DMSO ratios. (E) Retinoschisin accumulates within cells treated with 1 µM jasplakinolide. Cells were treated with DMSO or 1 µM jasplakinolide for 72 h. Cells were then fixed and incubated with RS24-37 antibody followed by Alexa Fluor 488 goat anti-rabbit IgG. Nuclei were labeled with DAPI.

We next assessed the effects of jasplakinolide, a natural toxin isolated from marine sponges that hyperpolymerizes the F-actin cytoskeleton, on retinoschisin secretion. Weri-Rb1 cells treated with 0.1 µM or 1 µM jasplakinolide were swollen and exhibited irregular morphology that was completely different from the smooth rounded shape of control cells or DMSO-treated cells (Fig. 4A). F-actin in untreated cells exhibited smooth fluorescence surrounding the cell surface. This fluorescence remained intact following 72 h exposure to DMSO, 0.001 µM or 0.01 µM jasplakinolide (Fig. 4A). On the other hand, 0.1 µM jasplakinolide caused diffused and reduced fluorescence throughout the cell cytoplasm and 1 µM jasplakinolide completely abolished phalloidin staining (Fig. 4A). This effect was the result of competitive binding of jasplakinolide to F-actin, which blocks the binding sites for phalloidin, as described by Bubb [26]. Retinoschisin secretion was remarkably reduced by approximately 70% with 1 µM jasplakinolide treatment (Figs. 4B and 4C). This concentration caused a 2.9±0.8 -fold accumulation of retinoschisin in the cells (Fig. 4D), suggesting that the F-actin cytoskeleton acts as a physical barrier to retinoschisin secretion.

**Figure 5:**
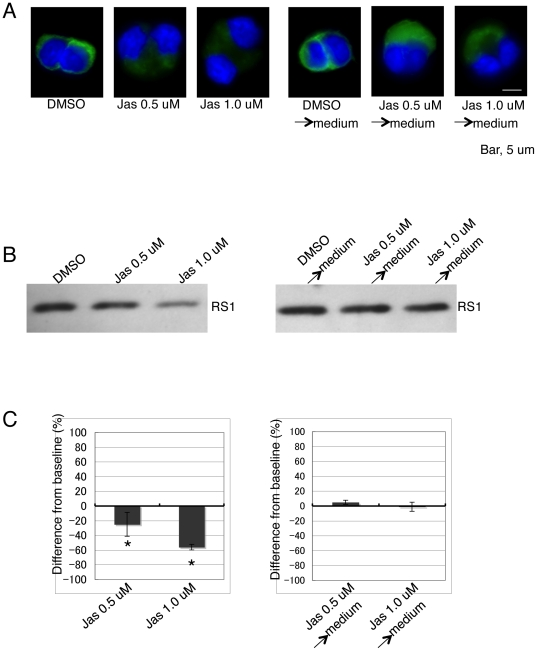
The effect of jasplakinolide on retinoschisin secretion is reversible. (A) The F-actin cytoskeleton was visualized by Alexa Fluor 488-phalloidin (green). Duplicate cell samples were treated with DMSO or 0.5 µM and 1.0 µM jasplakinolide (Jas) for 72 h and then one set was fixed and labeled for F-actin (left panel). The conditioned medium of the other set of samples was replaced by fresh medium without DMSO (DMSO→medium) or jasplakinolide (Jas 0.5 µM or 1.0 µM→medium) in it. Cells were incubated for another 72 h in the fresh medium, fixed, and labeled with Alexa Fluor 488-phalloidin. (B) Western blot of proteins from the medium of each sample described in (A). Retinoschisin was detected using the RS24-37 antibody. (C) Quantification of the intensity of each band on the Western blot in (B) (see MATERIALS AND METHODS). The data are means ± SEM of three different biological samples for each condition. *: p<0.05 was obtained for the difference between 0.5 µM or 1 µM jasplakinolide- and DMSO-treated cells.

To determine whether the inhibitory effect of jasplakinolide on retinoschisin secretion is reversible, the conditioned medium containing 0.5 µM or 1.0 µM jasplakinolide was replaced with toxin-free medium after 72 hr incubation and then the cells were further cultured for another 72 hr. The effect of jasplakinolide on retinoschisin secretion was completely reversed after removal of the toxin: bound jasplakinolide was released from F-actin, although the cell morphology and the F-actin structure were not fully recovered (Figs. 5A, B, C).

### Inhibition of ROCK changes F-actin structure and enhances retinoschisin secretion

**Figure 6:**
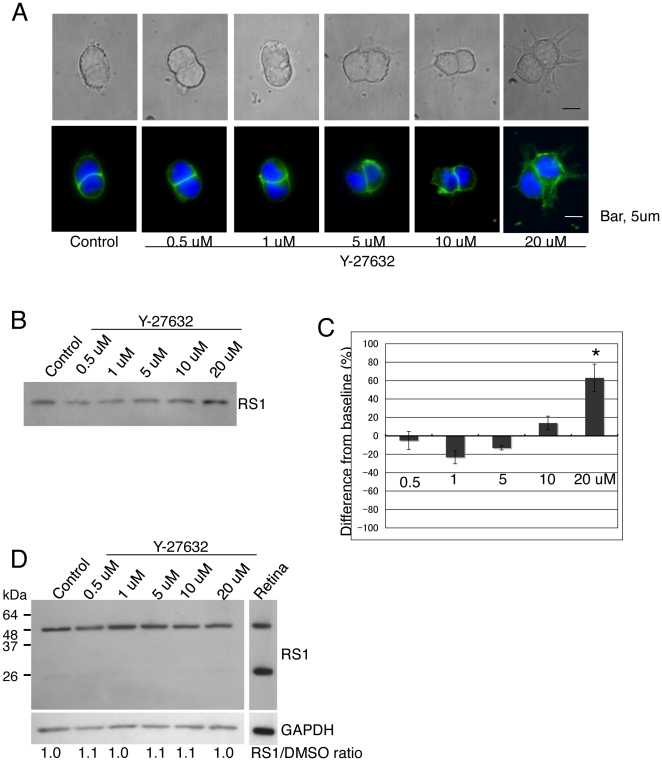
Retinoschisin secretion into the conditioned medium is increased by the ROCK kinase inhibitor, Y-27632. (A) Effects of Y-27632 on cell morphology and F-actin cytoskeleton organization. Weri-Rb1 cells were cultured in medium alone (untreated control) or the indicated concentrations of Y-27632 for 72 h. Cells were then fixed and labeled with Alexa Fluor 488-phalloidin (green). The upper row of pictures shows transmitted light images and the lower row, fluorescent light images. Bar, 5 µm. (B) Retinoschisin from the conditioned media of cells treated as in (A) was detected on a Western blot using the RS24-37 antibody. As seen, retinoschisin secretion into the conditioned medium is increased at 20 µM Y-27632. (C) Quantification of the intensity of each band on the Western blot from (B) (see MATERIALS AND METHODS). The data are means ± SEM of three different biological samples for each condition. *: p<0.05 was obtained for the difference between 20 µM Y-27632-treated and untreated cells. (D) The level of retinoschisin in the lysates of whole cells treated with the concentrations of Y-27632 used in (B) does not change. After 72 hr incubation in medium alone or medium containing the indicated concentrations of Y-27632, cells were collected and their retinoschisin was analyzed on a Western blot using the RS24-37 antibody. The band intensities of the dimer and monomer for each condition were added and normalized to GAPDH. The normalized values for treated and untreated cells were compared and are shown as RS1/control ratios.

Since it is well known that members of the family of small G-proteins regulate F-actin assembly [27], we investigated whether inhibition of the Rho-associated kinase (ROCK) of Weri-Rb1 cells by Y-27632, a highly specific Rho kinase inhibitor [28], could affect retinoschisin secretion. The effects of Y-27632 on cell morphology and the F-actin cytoskeleton were dose-dependent. Weri-Rb1 cells treated with 20 µM Y-27632 showed dramatic changes in morphology exhibiting spread cytoplasm and several long protrusions of phalloidin-stained F-actin (Fig. 6A). Smaller protrusions around cells were already seen at 10 µM Y-27632. At these concentrations, F-actin fluorescence was reduced and non-uniform throughout the cytoplasm. This is indicative of the F-actin cytoskeleton destabilization, resulting in the loosening or relaxing of F-actin fibers. Accordingly, treatment with 20 µM Y-27632 enhanced retinoschisin secretion by approximately 60% (Figs. 6B and 6C) and there was no accumulation of retinoschisin observed in cells (Fig. 6D). These data demonstrates that ROCK regulates the secretion of retinoschisin.

### DBcGMP enhances retinoschisin secretion

**Figure 7:**
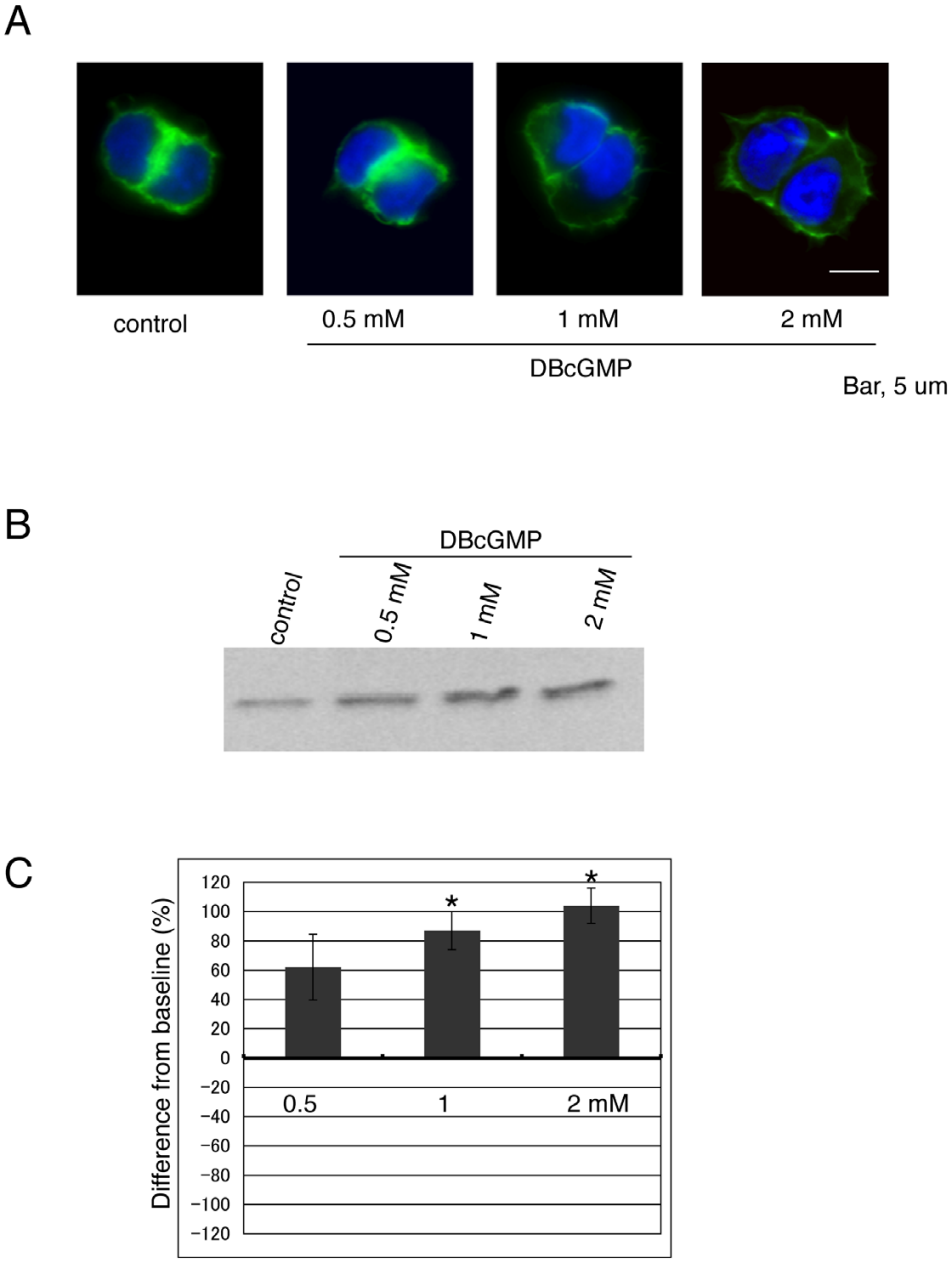
DBcGMP enhances retinoschisin secretion and modifies F-actin localization. (A) Effects of DBcGMP on the F-actin cytoskeleton. Weri-Rb1 cells were cultured with medium alone (untreated control) or the indicated concentrations of DBcGMP for 72 h. Cells were then fixed and labeled with Alexa Fluor 488-phalloidin (green). Note the F-actin protrusions in cells treated with 1 mM and 2 mM DBcGMP. Bar, 5 µm. (B) Retinoschisin secretion into the medium increases with the three concentrations of DBcGMP used to treat the cells for 72 h, as seen on the Western blot of the media samples reacted with the RS24-37 antibody. (C) Quantification of the retinoschisin bands on the Western blot in (B) (see MATERIALS AND METHODS). The data are means ± SEM of three different biological samples for each condition. Retinoschisin levels in the culture medium of cells treated with the different concentrations of DBcGMP are all higher than in the medium of untreated cells, but the results are statistically significant only for the cells treated with 1 and 2 mM DBcGMP. *: p<0.05 was obtained for the difference between DBcGMP-treated and untreated cells.

Several reports have shown that a cGMP-mediated pathway regulates F-actin cytoskeleton by inhibiting the RhoA/ROCK pathway [29]–[32]. DBcGMP, a permeable cGMP analogue, was employed to examine the involvement of cGMP in retinoschisin secretion from Weri Rb1 cells. At 1 mM DBcGMP, F-actin showed small protrusions around cells. The protrusions were extended at 2 mM DBcGMP and the phalloidin staining was reduced in the cytoplasm (Fig. 7A). These effects are similar to those produced by Y-27632 on Weri Rb1 cells (Fig. 6A). As seen in Figures 7B and 7C, treatment with 2 mM DBcGMP almost doubled retinoschisin secretion, suggesting the involvement of cGMP in this process.

### The microtubule-hyperpolymerizing reagent, taxol, suppresses retinoschisin secretion

**Figure 8:**
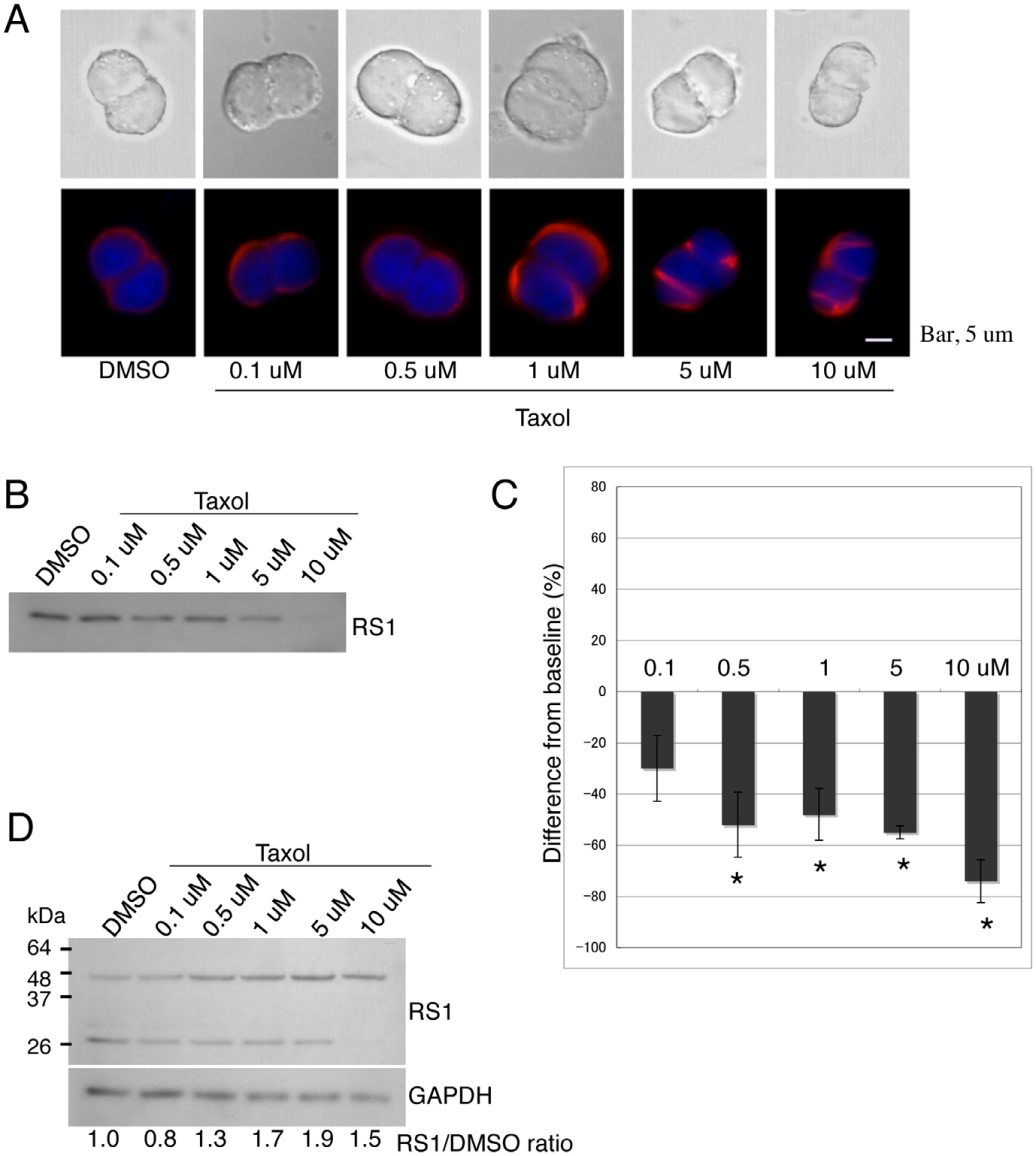
Retinoschisin secretion into the conditioned medium is decreased by addition of taxol. (A) Effects of taxol on cell morphology and microtubule organization. Weri-Rb1 cells were cultured with medium containing DMSO or the indicated concentrations of taxol for 72 h. Cells were then fixed and labeled with an α tubulin antibody (red). The upper and lower rows of pictures show transmitted light images and fluorescent light images, respectively. The microtubule cytoskeleton of cells shows bundles after treatment with 5 µM and 10 µM taxol. Bar, 5 µm. (B) Cells were treated with the indicated concentrations of taxol for 72 h and retinoschisin in the conditioned media was analyzed on Western blots using the RS24-37 antibody. Retinoschisin secretion into the medium decreases in a dose-dependent manner between 0.5 µM and 10 µM taxol. (C) Quantification of the intensity of the bands from the Western blot in (B) (see MATERIALS AND METHODS). The data are means ± SEM of three different biological samples for each condition. *: p<0.05 was obtained for the differences between 0.5 µM–10 µM taxol-treated and DMSO-treated cells. (D) After 72 hr incubation in medium containing DMSO or the indicated concentrations of taxol, cells were collected and analyzed by Western blot using the RS24-37 antibody. The intensities of the bands corresponding to the dimer and monomer of retinoschisin at each taxol concentration were added and normalized to GAPDH. The normalized values were compared to that of cells treated with DMSO and shown as RS1/DMSO ratios. Retinoschisin accumulates inside the cells exposed to taxol at concentrations between 0.5 µM and 10.0 µM.

Since the partial co-localization of retinoschisin and microtubules was observed in Figure 1, we investigated the involvement of the microtubule cytoskeleton in retinoschisin secretion using taxol, a modifier of microtubules that facilitates their hyperpolymerization [33]. At a concentration of 1 µM taxol, Weri Rb1 cells showed a thickened mass of microtubules, and at 5 µM taxol, disorganized bundles of microtubules were observed (Fig. 8A). Cells treated with 10 µM taxol were compressed and their microtubules were disorganized further. These observations indicated that the effect of taxol on the microtubule cytoskeleton was dose-dependent. Treatment of cells with 0.5 µM, 1 µM, or 5 µM taxol resulted in 50% reduction of retinoschisin secretion accompanied by accumulation of the protein within the cells (Figs. 8B and 8C). Incubation with 10 µM taxol reduced even more (approximately by 70%) secretion and the retinoschisin monomer was no longer present in the cells (Fig. 8D). These results suggest that the microtubule cytoskeleton also plays a role in retinoschisin secretion.

## Discussion

Ultrastructural studies of actively secreting cells have demonstrated the presence of extensive F-actin networks in their cytoplasm and a dense F-actin cortex under their plasma membrane [34]. This F-actin zone has been shown to be an obstacle that impedes exocytosis of secretory granules at the plasma membrane of neurons, neuroendocrine, endocrine, exocrine and hematopoietic cells [35]. Therefore, vesicular trafficking would require an active process of F-actin disassembly to mediate movement through cortical actin. However, it has been reported that the F-actin network plays both inhibitory and facilitatory roles in exocytosis [24]. On one hand, it acts as a physical barrier, preventing granule access to their appropriate docking and fusion sites on the plasma membrane, as proved by demonstrating that pharmacological disruption of F-actin enhanced basal and stimulus coupled exocytosis [34], [36], [37]. But on the other hand, the F-actin cytoskeleton also contributes to exocytosis, as shown by reduced exocytosis during pharmacological alteration of F-actin in some cell types [38]–[40]. Furthermore, active cytoskeletal rearrangements have been shown to accompany many vesicle transport and fusion events in a variety of cell systems [41], [42]. Our findings indicating that retinoschisin secretion is inhibited by actin depolymerizing (cytochalasin D) or hyperpolymerizing (jasplakinolide) drugs are consistent with secretion results reported for other proteins and cells in the literature [38], [40]. In addition, we observed accumulation of retinoschisin in Weri Rb1 cells treated with 2 µM cytochalasin D or 1 µM jasplakinolide. These results indicate that the F-actin cytoskeleton contributes to retinoschisin secretion but can also act as a barrier for its secretion.

It is important to note that low concentrations of cytochalasin D (0.2 µM) and jasplakinolide (0.1 µM) had no effect on retinoschisin secretion despite some changes exhibited on the F-actin cytoskeleton, whereas high concentrations of these drugs (2 µM and 1 µM, respectively) caused dramatic alterations of the F-actin cytoskeleton and suppressed retinoschisin secretion. Moreover, we showed that the effects of cytochalasin D and jasplakinolide on the secretion process were fully reversible upon removal of these drugs from the culture medium, even when complete restoration of the F-actin cytoskeleton was not achieved. These observations imply that F-actin must be present for retinoschisin secretion to occur.

Small GTPases of the Rho family are important regulators of many motile responses that involve the actin cytoskeleton and/or microtubule network [20]. Three main classes of Rho GTPases, Rho, Rac, and Cdc42, play key roles in vesicular secretion by regulating actin cytoskeletal dynamics [43], [44]. Furthermore, studies on different cell types have shown that inhibition of downstream effectors of Rho GTPases also contribute to vesicular trafficking by controlling the organization of the F-actin cytoskeleton. For example, inhibition of Rho's downstream effector, Rho-associated kinase (ROCK), and thereby of the Rho-ROCK signaling pathway, induces actin depolymerization and improves glucose-stimulated insulin secretion of primary pancreatic B-cells [45]. In mast cells, Rho controls F-actin disassembly and secretion [46]. In addition, late steps of exocytosis are affected as a consequence of actin filaments severed by gelsolin, a downstream effector of Rac [47]. In rabbit parietal cells, any treatment that inhibits Rho augments acid secretion, whereas those that activate Rho inhibit secretion via site-specific regulation of actin microfilaments [48]. In this study, we demonstrated that retinoschisin secretion was facilitated by the loosening or relaxing of the F-actin cytoskeleton through inhibition of ROCK with 20 µM Y-27632, confirming that the F-actin cytoskeleton functions as a physical barrier for retinoschisin secretion, as also demonstrated before by the accumulation of retinoschisin within cells treated with jasplakinolide.

Interestingly, we here demonstrate the involvement of cGMP in retinoschisin secretion. cGMP is an important second messenger that regulates a variety of intracellular activities, such as the contraction of cardiac and smooth muscle, the immune cell response, synaptic transmission, and secretion [49]–[51]. In retinal photoreceptors, cGMP mediates phototransduction, influx of Ca^2+^, transmitter release, and exocytosis [52]–[54]. In this paper, we show that DBcGMP, a permeable analogue of cGMP, altered F-actin structure and enhanced retinoschisin secretion, causing similar results to those obtained when Weri RB1 cells were treated with the ROCK inhibitor Y-27632. In fact, a cGMP-mediated pathway is known to inhibit RhoA/ROCK and control cytoskeleton dynamics [29], [31], suggesting that the RhoA/ROCK pathway might regulate retinoschisin secretion. This finding unveils a new function for cGMP in retina.

Microtubules are dynamic cytoskeletal components that are important for the maintenance of cell shape and polarity, in secretion, signaling, migration, and other cellular processes [55]. Taxol is a potent inhibitor of eukaryotic cell proliferation. It binds reversibly along the surfaces of microtubules, and at high concentrations (1–20 µM) it enhances microtubule polymerization and also induces the formation of extensive bundles of microtubules in cells. In human cancer cells, low concentrations of taxol (<0.1 µM) block or slow mitosis and suppress microtubule dynamic instability and treadmilling without changing the overall microtubule polymer mass [55]. In this study, a low concentration of taxol (0.1 µM) had no effect on the microtubule network of Weri Rb1 cells or on retinoschisin secretion. In contrast, concentrations of taxol higher than 1 µM resulted in dramatic alterations of microtubule structure, such as thickened microtubules and disorganized bundles, and caused a decrease in retinoschisin secretion. These results are consistent with those of other studies that showed that taxol inhibits microtubule-dependent secretion or vesicle transport in cultured cells [56], [57]. Since it has been described that microtubules act as tracks to transport vesicles [58], it is possible that the taxol-thickened and bundled microtubules that we observe may not be able to function as tracks, leading to the reduction of retinoschisin secretion and accumulation of the protein in Weri Rb1 cells. Thus, our experiments suggest that an intact microtubule cytoskeleton is required for retinoschisin secretion, and that microtubules may indeed act as tracks for retinoschisin leading the protein to the cells' plasma membranes. However, we observed that 0.5 µM taxol suppressed retinoschisin secretion without significant microtubule alteration. A reasonable explanation for this could be that taxol induces fragmentation of the trans-Golgi network (TGN), as previously reported [59], and thus it may disturb the sorting or packaging of proteins at the TGN with a concomitant decrease in their secretion.

**Figure 9:**
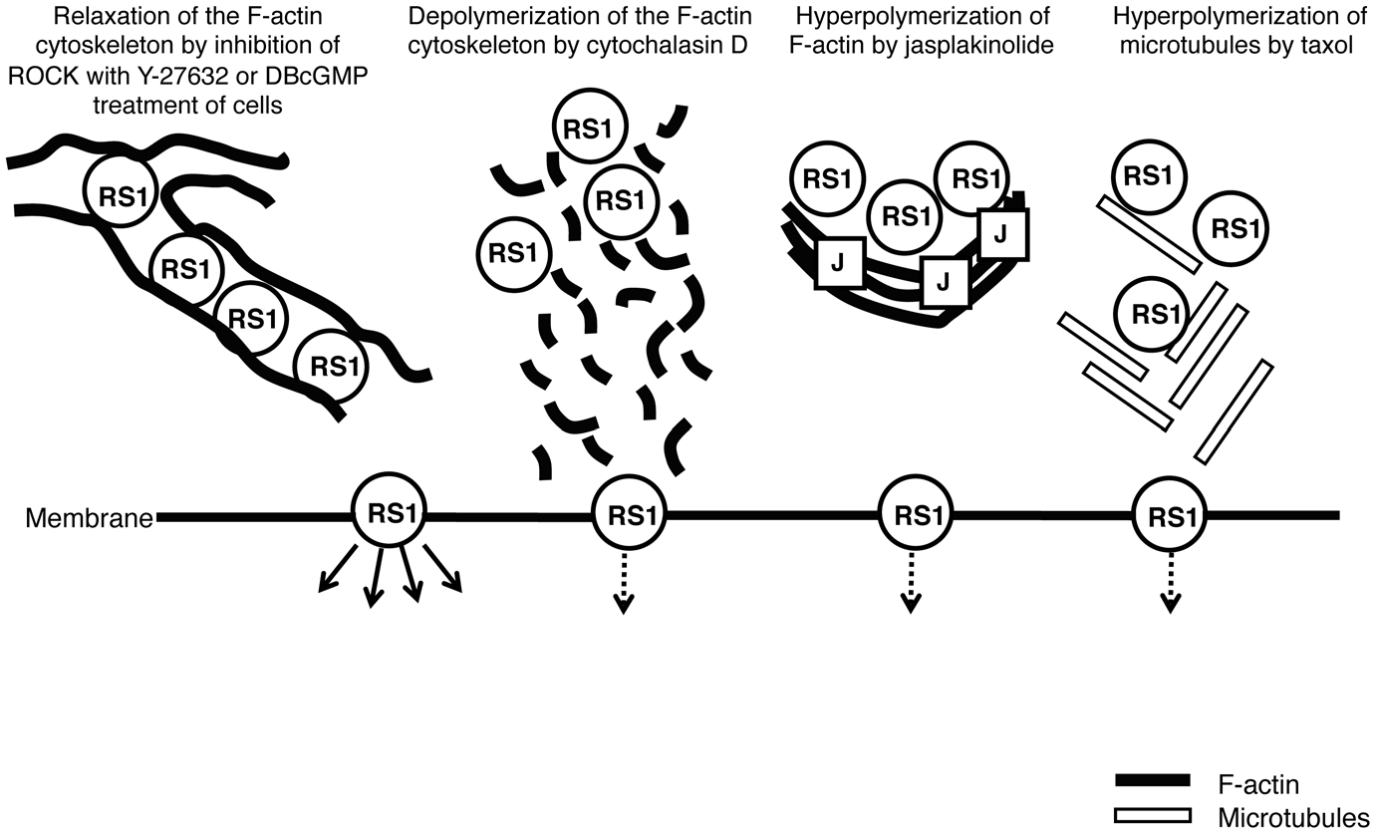
Summary diagram for the roles of the F-actin and microtubule cytoskeletons in retinoschisin secretion. Different pharmacological treatments of Weri-Rb1 cells showed that: The F-actin depolymerizing drug cytochalasin D causes fragmentation of F-actin and inhibits secretion of retinoschisin in a dose-dependent manner at concentrations higher than 0.5 µM. At 2 µM cytochalasin D, this inhibition of secretion results in a 2.1-fold accumulation of retinoschisin within the cultured cells. The natural toxin Jasplakinolide (1 µM) hyperpolymerizes F-actin and results in the reduction of retinoschisin secretion and its 2.9-fold accumulation within the cells. Inhibition of ROCK using 20 µM Y-27632 or treatment with 2 mM DBcGMP relaxes the F-actin cytoskeleton and almost doubles retinoschisin secretion. Hyperpolmerization of microtubules by taxol results in disorganized microtubule bundles that reduce retinoschisin secretion. The solid arrows indicate increased retinoschisin secretion and the dotted arrows, decreased secretion.

In summary, we showed that retinoschisin secretion is highly regulated by cytoskeleton structures and by a signaling pathway: a dual function of the F-actin cytoskeleton can both hinder and mediate retinoschisin secretion, a cGMP-mediated inhibition of ROCK is possibly involved in the regulation of this process and the microtubule cytoskeleton is required for retinoschisin secretion to occur, as depicted in Figure 9.

Recently it was demonstrated that retinoschisin secretion is under circadian control [17] and that L-type voltage-gated calcium channels (L-VGCCs) are involved in this control [60]. Interestingly, it has been reported that one of the small GTPases, Rab3A, influences circadian rhythms in the mouse [61]. Evidence implicating Rab3A in exocytosis has also been described for different cells including chromaffin cells, PC12 cells, insulin secreting cells, and pancreatic exocrine cells [35]. It is possible that Rab3A may play a key regulator role in the circadian control of retinoschisin secretion. Moreover, a subset of Rab and SNARE proteins, which are implicated in vesicle trafficking and membrane fusion, were identified in retina outer segments by a tandem mass spectrometry-based proteomics approach [62]. Further studies are needed to define the function of various small GTPases and proteins involved in cellular signal transduction as regulators of intracellular cytoskeleton dynamics. The results of these studies will give greater insight into the molecular mechanisms underlying retinoschisin secretion and eventually lead to the development of treatment strategies for XLRS.

## Materials and Methods

### Secretion experiments and chemical reagents used

Weri-Rb1 human retinoblastoma cells [21] were obtained from the American Type Tissue Culture Collection (Manassas, VA) and cultured in RPMI-1640 medium (Thermo Scientific HyClone, Logan, UT) supplemented with 10% fetal bovine serum (Hyclone) at 37°C in 5% CO2. For secretion experiments, cells were plated on six-well dishes (3×10^6^ cells per well) coated with poly-D-lysine (0.2 mg/ml, Sigma-Aldrich, St. Louis, MO) and fibronectin (5 µg/ml, Sigma-Aldrich). Cells were grown in 1 ml of medium containing different concentrations of drugs. Drugs used were: cytochalasin D and jasplakinolide [EMD4 Biosciences (Calbiochem, San Diego, CA)], and paclitaxel (taxol), Y-27632 and dibutyryl cGMP (DBcGMP) (Sigma-Aldrich). Cytochalasin D, jasplakinolide, and taxol were dissolved in dimethylsulfoxide (DMSO). DMSO concentration did not exceed 1% in the cultures. Cells were cultured for 72 h and their protein extracts were subjected to Western blot analysis using RS24-37, an affinity purified rabbit polyclonal antibody previously generated against a synthetic peptide corresponding to amino acid residues 24-37 of retinoschisin [15] (ProSci Inc., San Diego, CA).

### Western blot analysis

After 72 h incubation with each drug, conditioned media and cells were collected separately. The conditioned media were filtered using an Ultrafree-MC filter (0.1 µm pore size, Millipore, Billerica, MA) to remove cell debris. Cells were incubated on ice for 15 min with lysis buffer containing 50 mM Tris-HCl (pH 7.5), 150 mM NaCl, 1 mM EDTA, 1 mM EGTA, 1% Triton X-100, 1 mM PMSF, and Halt Protease Inhibitor Cocktail (Thermo Scientific Pierce, Rockford, IL) and then the cell debris was cleared by centrifugation at 10,000 g for 20 min at 4°C. The filtered conditioned media or the cell lysates were incubated in a denaturing solution (100 mM Tris, pH 6.8, 1% SDS, 10% glycerol, and 90 mM DTT) at room temperature for at least 1 h and resolved on 10% polyacrylamide SDS gels (BioRad, Hercules, CA). Proteins were electroblotted onto PVDF membranes (BioRad) in transfer buffer (50 mM Tris, 380 mM glycine, and 20% methanol) overnight at 4°C. The membranes were blocked using 3% nonfat milk in TBST (50 mM Tris, pH 7.4, 500 mM NaCl, and 0.1% Tween 20) and incubated with RS24-37 (1∶400) or with monoclonal anti-GAPDH (1∶10,000, clone 6C5, Applied Biosystems, San Diego, CA) containing 3% BSA in TBST, for at least 1 hr. After incubation with antibodies, the membranes were washed three times in TBST, incubated with an alkaline phosphatase-conjugated goat-anti-rabbit IgG or horse-anti-mouse IgG, respectively (Vector Laboratories, Burlingame, CA) for 1 h, and washed three times in TBST. Bands were detected on Amersham Hyperfilm-MP (GE Healthcare UK Ltd., Piscataway, NJ) by using the DuoLux chemiluminescence substrate (Vector Laboratories). The density of the bands was quantified using a ChemiImager 5500 (Alpha Innotech, San Leandro, CA). The amount of retinoschisin in the culture medium of the control sample (RPMI 1640 medium or RPMI 1640 medium containing only DMSO) was used as baseline and it was measured in every blot of each experiment. The level of retinoschisin in each conditioned medium was estimated as the percent of the difference from the baseline on the same blot. The density of retinoschisin from cells was normalized to that of GAPDH on the same blot. Paired *t*-test (two-tailed) was employed for comparisons between 2 groups. A value of p<0.05 was considered statistically significant.

### Immunocytochemistry

Cells were plated onto cover slips coated with poly-D-lysine (0.2 mg/ml) and fibronectin (5 µg/ml) in twelve-well dishes (2×10^5^ cells per well). After 72 h incubation with each drug, cells were washed twice with PBS, fixed with 4% paraformaldehyde for 10 min at room temperature and blocked by incubation for 1 h in PBS containing 1% BSA, 5% normal serum, and 0.1% Triton X-100. Primary antibodies used were RS24-37 (1∶50) and monoclonal anti-alpha tubulin (1∶250; DM1A, eBioscience, San Diego, CA). Secondary antibodies used were indocarbocyanine (Cy3)-conjugated donkey-anti-rabbit IgG (for staining retinoschisin red, 1∶200, Jackson ImmunoResearch Laboratories, Inc., West Grove, PA); Alexa Fluor 488 goat anti-rabbit IgG (for staining retinoschisin green) and Alexa Fluor 568 goat anti-mouse IgG (for staining tubulin red), both at dilutions of 1∶1000 (Invitrogen-Molecular Probes, Carlsbad, CA). F-actin was visualized by staining with Alexa Fluor 488-phalloidin (Invitrogen-Molecular Probes, 1∶100). Cells were washed with PBS containing 0.1% Tween 20 (4 times, 5-min each) following incubation with primary and secondary antibodies. After immunostaining, samples were mounted on slides using Biomeda Gel-Mount medium (Electron Microscopy Sciences, Hatfield, PA) and analyzed by confocal microscopy (Leica TCS SP2 microscope, Leica Microsystems, Germany) or a fluorescence microscope (Axioplan, Carl Zeiss, Germany).
